# Phytochemical profile and antimicrobial activity of the leaves and stem bark of *Symphonia globulifera* L.f. and *Allophylus abyssinicus* (Hochst.) Radlk

**DOI:** 10.1186/s12906-022-03692-0

**Published:** 2022-08-23

**Authors:** Ben Lukubye, Clement Olusoji Ajayi, Rapheal Wangalwa, Grace Kagoro-Rugunda

**Affiliations:** 1 Department of Biology, Mbarara University of Science and Technology, P.O. Box 1410, Mbarara, Uganda; 2 Department of Pharmacy, Mbarara University of Science and Technology, P.O. Box 1410, Mbarara, Uganda

**Keywords:** *Allophylus abyssinicus*, *Antimicrobial activity*, *Combination* effect, *Symphonia globulifera*, Phytochemical profile

## Abstract

**Introduction:**

*Symphonia globulifera* and *Allophylus abyssinicus* are used in the management of skin rashes and sores, cough, malaria, digestive diseases, stomach ache, wounds and helminthic infections among others in Uganda, Kenya, Ethiopia, Cameroon. This study aimed at determining the phytochemical profile and antimicrobial activity of these two plants.

**Methods:**

The stem bark and leaves of both plants were collected from Bwindi Impenetrable National Park and air-dried under shade at room temperature. Cold maceration, decoction and infusion with methanol, water and ethyl acetate as solvents were used in phytochemical extraction. Preliminary qualitative screening and thin layer chromatography were used for phytochemical profiling. Antimicrobial activity was analysed by agar well diffusion assay, broth macro-dilution assay and fractional inhibition concentration index (FICI).

**Results:**

The leaves and stem bark of both plants have a diverse set of phytochemical compounds of variable polarity including, tannins, alkaloids, flavonoids, saponins, quinones and anthraquinones among others. Generally, methanol and water extracts of *S. globulifera* and *A. abyssinicus* had *in-vitro* bactericidal activity against *Staphylococcus aureus, Escherichia coli* and *Pseudomonas aeruginosa* but weak fungistatic activity against *Candida albicans*. *Allophylus abyssinicus* leaf water and *S. globulifera* leaf methanol extract combination had a synergistic activity (ΣFICI = 0.37) against *S. aureus*. Similarly, *A. abyssinicus* stem bark water extract and *A. abyssinicus* leaf water extract combination had an additive effect (ΣFICI = 1) against *P. aeruginosa*.

**Conclusion:**

The leaves and stem bark crude extracts of *S. globulifera* and *A. abyssinicus* possess a wide range of bioactive phytochemical compounds but have weak antimicrobial activity against *S. aureus*, *E. coli, P. aeruginosa* and *C. albicans.*

## Introduction

The usage of natural products of plant or animal origin for medicine dates back to prehistoric times at least 60,000 years ago [1]. Currently, 149 (88%) of the 170 member states of the world health organization (WHO) acknowledge the usage and regulation of traditional medicine including herbal medicines for primary health care [2] greatly higher than the 64 (37%) member states as of the year 2000 [3]. This could indicate the growing popularity and usage of traditional medicine among the people. The past two decades have witnessed an incredible increase in acceptance and public interest in natural therapies both in developing and developed countries. This is attributed to several factors including, limited availability and accessibility of the pharmaceutical products, high prices of the drugs, side effects associated with the pharmaceutical drugs and the society’s growing general disapproval of the modern pharmaceutical drugs [4]. In developing countries, the limited number of modern health care workers has contributed greatly to the continued usage of traditional medicine, for instance, the ratio of traditional healers to the population in Africa is 1:500 whereas the ratio of medical doctors to the population is 1:40,000 [5]. Due to this, the WHO has advocated for the incorporation of herbal medicine into the public health care systems of the different countries through authentication of the herbal extracts using modern scientific techniques. With the rapidly growing drug resistance especially among microorganisms, plants are potential sources of novel antibiotics and drugs but only 15% of the world’s higher plants had been systematically investigated for bioactivity [6].

*Symphonia globulifera* L.f. belongs to a large family Clusiaceae and is widely distributed along the swamps of low land tropical rainforests of tropical countries like Madagascar, Cameroon, Uganda, Gabon, Nigeria, Panama, Brazil and Colombia [7]. In Uganda, *S. globulifera* mainly occurs in the Rwenzori mountains and Sango bay area [8, 9]. *Symphonia globulifera* L.f. has been used to treat a wide range of diseases including skin rashes and sores, cutaneous Leishmaniasis, cough, malaria, digestive diseases, diarrhea and stomach ache [7, 9, 10]. This shows the antimicrobial and antiparasitic potential of this plant. Interestingly in vitro antiparasitic studies have shown the potential of extracts and isolated compounds from the stem bark *S. globulifera* to significantly inhibit the growth of *Plasmodium falciparum*, *Trypanosoma brucei* and *Leishmania donovani* with the activity attributed to benzophenones and xanthones [10–12]. However, the phytochemical profile and the antimicrobial activity of the leaves and stem bark of this potent medicinal plant have not been investigated hence the focus of the current study.

On the other hand, *A. abyssinicus* (Hochst.) Radlk. is a medium to large-sized tree that belongs to the family *Sapindaceae* and a large genus *Allophylus* with about 255 species distributed worldwide in the tropical and subtropical regions [13]. The leaves of *A. abyssinicus* are used in the management of helminthic infections, sexually transmitted diseases, boils [14] and skin diseases in Ethiopia [15]. The bark is used for the treatment of diarrhea, and wounds in Ethiopia [16]. The roots of *A. abyssinicus* are used in the treatment of cough in Kenya. The value of this plant is not restricted to humans alone in that the leaves of *A. abyssinicus* are often eaten by mountain gorillas in the Bwindi forest impenetrable national park hence hypothesized to be an anthelmintic self-deworming avenue [17]. Despite the above-mentioned medicinal uses of  *A. abyssinicus*, only the anti-inflammatory and wound healing activity of the plant have been investigated with results showing genuine wound healing activity [14]. However, no study has been carried out to investigate the phytochemical profile and the antimicrobial activity efficacy of *A. abyssinicus* stem bark and leaf extracts.

Determination of the phytochemical profile of extracts from *S. globulifera* and *A. abyssinicus* plant parts is key to the development of a monograph for usage in the incorporation of herbal medicine into modern public health care systems. The study aimed at assessing the phytochemical composition and microbial activity of *S. globulifera* and *A. abyssinicus.* We hypothesized that there is no significant difference in the microbial activity of the stem bark and leaf extracts of *S. globulifera and A. abyssinicus* on selected microbial strains *(S. aureus*, *E. coli, P. aeruginosa* and *C. albicans).*

## Materials and methods

### Plant material collection area

**Fig. 1:**
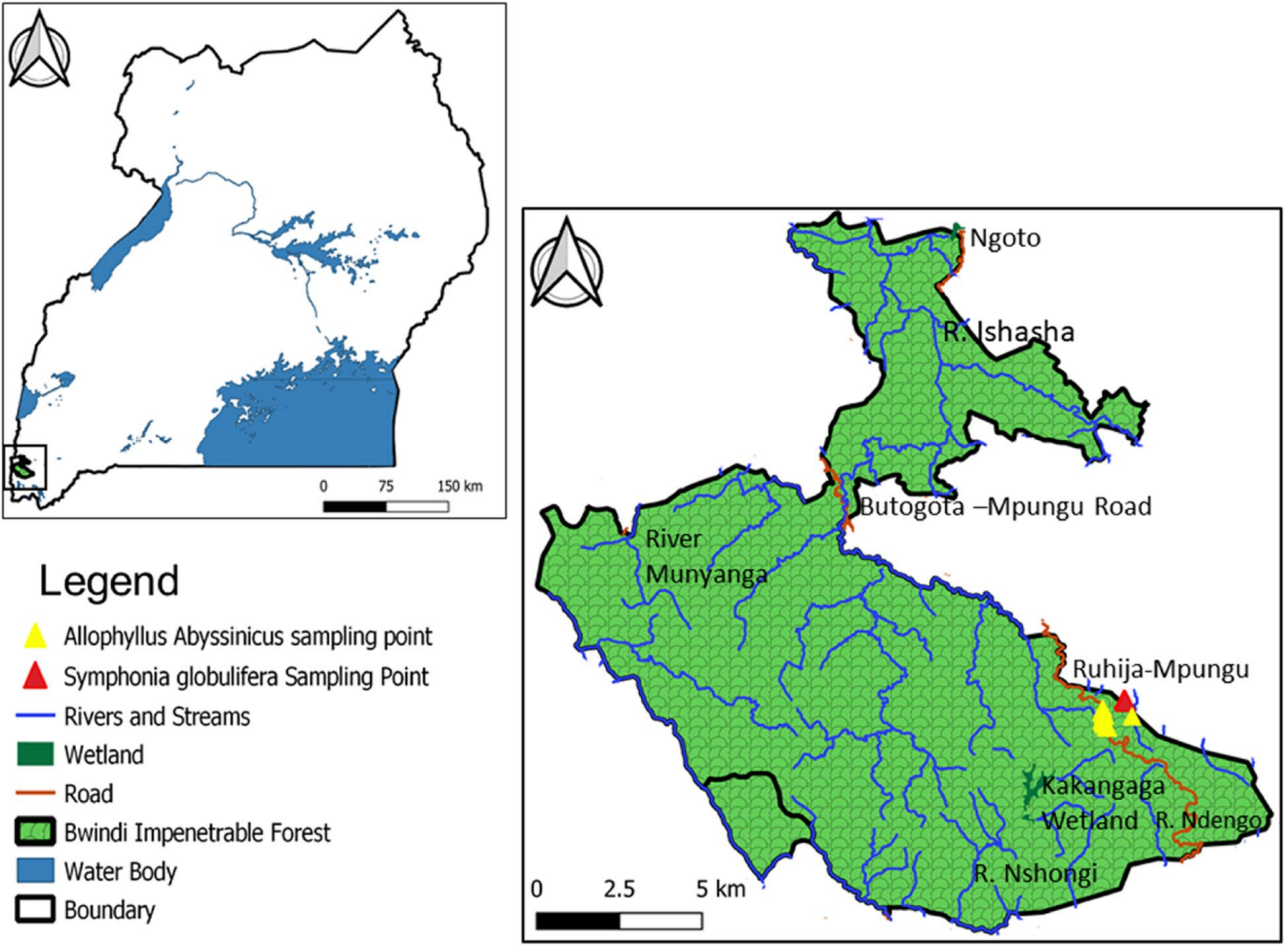
A map of Uganda showing location of Bwindi Impenetrable Forest National Park and plant sample collection sites

The leaves and stem bark of both *S. globulifera* and *A. abyssinicus* were collected from the Ruhija sector, Bwindi Impenetrable National Park (BINP), located in south western Uganda at coordinates between latitude 0°53’S and 1°8’S and between longitude 29°35’E and 29°50’E on the eastern edge of the Albertine rift valley [18] as shown in Fig. 1 below. Permission to access the Bwindi Impenetrable National Park for sample collection and transportation was obtained from the Uganda Wildlife Authority (UWA) under reference number COD/96/05. Bwindi Impenetrable National Park was chosen purposively as the source of samples because of the availability of the selected plants but also due to the great conservation efforts.

### Plant material collection

*Symphonia globulifera* and *A. abyssinicus* plants were identified with the help of a field botanist from the Institute of Tropical Forest Conservation (ITFC) from a highly conserved area with no or limited human interference. Mature healthy plants were selected as sampling units [19, 20]. The stem bark was harvested using the vertical alternate strip bark two-quarters technique [19, 21, 22]. Pruning shears were used to cut the leaves from the same trees from which stem bark was obtained. Furthermore, confirmation of the plant identity was performed by Dr. Eunice Apio Olet Alele (plant specialist, Mbarara University of Science and Technology). Herbarium specimen were submitted to the national herbarium at Makerere University under accession numbers MHU51049 and MHU51050 for *S. globulifera* and *A. abyssinicus* respectively.

### Preparation of extracts

Air-dried leaves and stem bark of *S. globulifera* and *A. abyssinicus* were independently crushed into powder using an electric grinder and extracted using water, methanol (70%) and ethyl acetate (100%) as solvents. Cold maceration was used for the extraction using ethyl acetate (100%) and methanol (70%) as solvents with an extraction time of 24 h and 48 h respectively. The decoction technique was used in the extraction of phytochemicals from the stem bark of the two plants with water as a solvent for 15 min. The infusion technique was used in the extraction of phytochemicals from the leaves with water as a solvent for 45 min [23, 24]. The extracts were filtered using the Whatman No. 1 filter paper and the filtrate concentrated using a Rotary evaporator at 70 ^∘^C and air-dried. Air-dried extracts were placed into tightly stoppered sterile glass bottles, labelled and then stored in a refrigerator at 4 °C for further analysis.

### Phytochemical screening

Qualitative phytochemical screening for saponins, alkaloids, tannins, flavonoids, terpenoids, cardiac glycosides, phlobatannins, anthraquinones, steroids, phenols, anthocyanins, quinones and chalcones in the methanol, water and ethyl acetate extracts were done following standard methods as described by [25–31].

### Phytochemical profiling using Thin Layer Chromatography (TLC)

Plant extracts were dissolved in their respective extraction solvents and spotted onto the TLC Analytica plate (20 × 20 cm) and left to dry. The TLC plate was then placed into the TLC tank with Hexane, Chloroform and Methanol 6:3:1 (v/v) as the solvent system and monitored for 2 h. The different phytochemical spots were visualized under normal light and ultraviolet (UV) light at 254 nm and 365 nm separately. For visualization of phenolic compounds, the TLC plate was sprayed with concentrated sulphuric acid and then heated for 4 min for better visualization [25, 32]. The retention factor of the different components (R_f_) was computed using the formula below.

$$\textit{Retention Factor (Rf)}=\frac{\text{Distance moved by the spot (cm)}}{\text{Solvent Front (cm)}}$$

### Antimicrobial activity of individual plant extracts and extract combinations

Plant individual extracts namely: *S. globulifera* leaf water extract (SGLW), *A. abyssinicus* leaf water extract (AALW), *A. abyssinicus* stem bark water extract (AASW) and *S. globulifera* leaf methanol extract (SGLM) and plant extract combinations including (SGLW and AALW, SGLW and SGLM, AASW and AALW, SGLM and AASW, SGLW and AASW, AALW and SGLM) were investigated for antimicrobial activity and their combination effect. For each extract combination for example SGLW and AALW, 1 g of each extract was measured separately into the same sample bottle and dissolved to make the extract combination sample.

Standards stock cultures of *Staphylococcus aureus* ATCC 25923, *Escherichia coli* ATCC 25922 and *Pseudomonas aeruginosa* ATCC 27853 and *Candida albicans* ATCC 10231 were obtained from the Microbiology laboratory, Mbarara University of Science and Technology. The bacteria strains were sub-cultured on Mueller Hinton Agar (MHA) and incubated at 37 °C for 48 h. *Candida albicans* ATCC 10231 was sub-cultured on Sabouraud Dextrose (SDA) agar at 37 °C for 72 h. Fresh sub-cultures were used in the antimicrobial activity assays as described below.

#### Agar well diffusion assay

Agar well diffusion assay [33, 34] was used to determine the zone of inhibition for the different plant extracts and extract combinations. Plant extract stock solutions (1000 mg/ml) were prepared by dissolving, 2 g of the extract or combination into 2 ml of water for water extracts or DMSO (1%) for methanolic extracts and consequently diluted further to obtain doubling dilutions 500 mg/ml, 250 mg/ml, 125 mg/ml, 62.5 mg/ml, 31.25 mg/ml, 15.625 mg/ml, 7.8125 g/ml, 3.9062 mg/ml and 1.9531 mg/ml. The test microorganism was inoculated onto the surface of the sterile MHA (bacteria strains) and SDA (*C. albicans*). Using a micropipette, 200 μl of plant extract and extract combination solutions of known concentration (1000 mg/ml, 500 mg/ml, 250 mg/ml, 125 mg/ml, 62.5 mg/ml, 31.25 mg/ml, 15.625 mg/ml, 7.8125 g/ml, 3.9062 mg/ml and 1.9531 mg/ml) were put into different wells (8 mm in diameter) on freshly prepared sterile MHA and SDA plates. Fluconazole (2 mg/ml) and 200 μl of Ciprofloxacin (1 mg/ml) were used as positive controls for the anti-fungal and antibacterial assays respectively. Furthermore, 200 μl of DMSO (1%) or distilled water was placed into a free well as a solvent control and an empty well was left to act as a negative control in each assay. The plates were then incubated at 37 °C for 24 h for antibacterial activity assays and at 37 °C for 72 h for anti-fungal activity assays. After incubation, the diameter of the zone of inhibition was then measured using a ruler. The experiments were repeated in quadruplicates.

#### Minimum Inhibitory Concentration (MIC)

The MIC of extracts and extract combinations were determined using the broth macro dilution method [35–37]. Stock plant extract or combination solution (1 g/ml) was prepared as described above. Stock extract or combination solution (1 ml) was placed into a Bijou bottle with Brain heart infusion broth (1 ml) to make a concentration of 500 mg/ml and then diluted into doubling dilutions using brain heart infusion broth to make extract concentrations of 500 mg/ml, 250 mg/ml, 125 mg/ml, 62.5 mg/ml, 31.25 mg/ml, 15.625 mg/ml, 7.8125 g/ml, 3.9062 mg/ml and 1.9531 mg/ml respectively in different Bijou bottles. These were each inoculated with 100 µl of 1.5 X 10^8^ (CFU/ml) of a specific test microorganism (*S. aureus, E. coli, P. aeruginosa* and *C. albicans*). Bijou bottles inoculated with bacteria (*S. aureus, E. coli, P. aeruginosa)* and *Candida albicans* were incubated at 37 °C for 24 h and at 37 °C for 72 h. Bijou bottles were removed and visually inspected for turbidity and growth. MIC was defined as the lowest extract concentration at which there was no visible growth. The above procedure was repeated in duplicates. The media, microorganism viability and negative control were done for each assay.

#### Minimum bactericidal concentration and Minimum fungicidal concentration

Bacterial and fungal inoculum within Bijou bottles with no visible growth after determination of the MIC were sub-cultured separately on freshly prepared MHA and SDA respectively and incubated at 37 °C for 48 h for bacteria and incubated at 37 °C for 72 h for *C. albicans*. The lowest concentration of the plant extracts that did not yield any colony on the solid agar after incubation was taken as the Minimum Bactericidal Concentration (MBC) and Minimum Fungicidal Concentration (MFC). The presence of growth from all sub-cultured doubling dilutions was indicative of fungistatic or bacteriostatic activity [38]. The above procedure was repeated in duplicates.

Total antibacterial activity and total antifungal activity (TAA) were calculated following a standard formula [39, 40].

$$TAA(ml/g)=\frac{\textit{Mass of extract from}\text{ 1 }\textit{gram of powder}\text{ (}\textit{mg per gram}\text{)}}{MIC\;(mg\;per\;ml)}$$

The combination effect of the selected plant extracts on the selected microbial strains was studied using fractional inhibitory concentration index (ΣFIC) [41, 42]. The fractional inhibitory concentration (FIC) for each extract was calculated using the formulae below.

$${FIC}^{B}= \frac{\mathrm{MIC }\left(\mathrm{A}+\mathrm{B}\right)\mathrm{combination}}{MIC (B)\mathrm{Independently}}$$

$${\mathrm{FIC}}^{\mathrm{A}}= \frac{\mathrm{MIC }\left(\mathrm{A}+\mathrm{B}\right)\mathrm{ combination}}{\mathrm{MIC }\left(\mathrm{A}\right)\mathrm{ Independently}}$$

where A and B represent different plant extracts.

The fractional inhibitory concentration index (ΣFIC) was then calculated as the sum of the fractional inhibitory concentration (FIC) of both extracts using the formulae below

$$\mathrm{\Sigma FIC}={FIC}^{A }+ {FIC}^{B}$$

Combination effect was interpreted following the fractional inhibitory concentration index scale i.e. ΣFIC =  ≤ 0.5 was considered as synergistic interaction, and ΣFIC =  > 0.5–1.0 was considered as additive interaction and ΣFIC > 1.0– ≤ 4 was considered as noninteractive and ΣFIC > 4.0 was considered as antagonistic [41].

### Data analysis

The data was analysed for descriptive statistics and presented as mean ± SD in form of tables. One-way ANOVA and LSD Post-hoc tests were used to analyse for statistical differences in the mean zones of inhibition of the different extracts and combinations against the test microorganisms. All statistical tests were carried out at a 5% level of significance in Statistical Package for the Social Sciences (SPSS) version 23.

## Results and discussion

### Percentage extract yield of *Symphonia globulifera* and *Allophylus abyssinicus* extracts

**Fig. 2:**
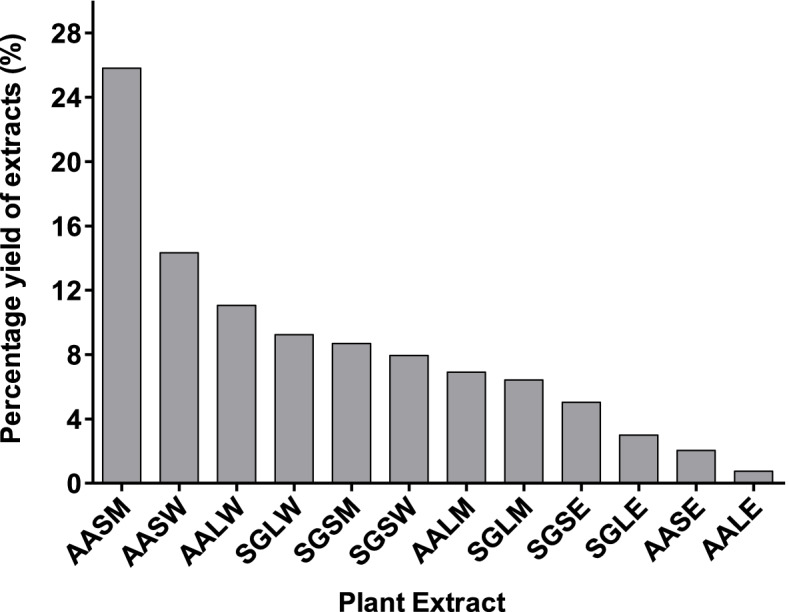
Percentage Yield of Extracts. Key: AASM:-*Allophylus abyssinicus* Stem Bark Methanol Extract, AASW:- *Allophylus abyssinicus* Stem Bark Water Extract, AALW:- *Allophylus abyssinicus* Leaf Water Extract, SGLW:-*Symphonia globulifera *Leaf Water extract, SGSM:-*Symphonia globulifera* Stem Bark Methanol extract, SGSW:- *Symphonia globulifera* Stem Bark Water extract, AALM:-*Allophylus abyssinicus* Leaf Methanol Extract, SGLM:-*Symphonia globulifera* Leaf Methanol extract, SGSE:- *Symphonia globulifera* Stem Bark Ethyl Acetate extract, SGLE:-* Symphonia globulifera *Leaf Ethyl Acetate extract, AASE:-*Allophylus abyssinicus* Stem Bark Ethyl Acetate Extract, AALE:-*Allophylus abyssinicus* Leaf Ethyl Acetate Extract

The percentage yield of methanol, water and ethyl acetate extracts from the stem bark and leaves of *S. globulifera* and *A. abyssinicus* ranged from 0.75% to 25.82% with *A. abyssinicus* stem bark methanol extract (AASM) showing the highest percentage yield (25.82%) as shown in Fig. 2. Generally, ethyl acetate showed the lowest extraction capacity (2.99%, 0.75%, 2.04% and 5.04%) compared to methanol and water for both leaves and stem bark. The difference in the percentage yield of the extracts is mainly caused by the difference in polarity of the extracting solvent. Polar solvents like water have a high affinity for polar compounds compared to non-polar bioactive compounds [43]. The observed higher percentage yield of water and methanolic leaf and stem bark extracts from both plants in this study indicates that polar phytochemicals and bioactive compounds are readily available in the leaves of these plants compared to the non-polar compounds. Methanol has also demonstrated a higher extraction percentage yield among other plants like *Severinia buxifolia* (Rutaceae) [44].

### Phytochemical profile of *Symphonia globulifera* and *Allophyllus abyssinicus*

Qualitative screening showed a wide range of phytochemical compounds including saponins, steroids, terpenoids, alkaloids, flavonoids, tannins, phenol, polyphenols and quinones among others occurring in higher amounts within one of the extracts of the two plants as shown in Table 1. Tannins, polyphenols and phenols were present in all methanol, ethyl acetate and water extracts of both *S. globulifera* and *A. abyssinicus.* However, saponins were only present in the methanol and water extracts of both extracts but absent in all the ethyl acetate extracts of the two plants. The phytochemical profile in any plant extract is significantly affected by the type of solvent due to differences in the polarity of the different phytochemical compounds [45]. Therefore, extraction solvents should always be selected carefully considering the polarity of the targeted bioactive compounds and that of the solvent.

**Table 1:** Phytochemical screening of the leaves and stem bark extracts of *Symphonia globulifera* and *Allophylus abyssinicus*

Phytochemical Class	SGSM	AASM	SGSE	AASE	SGSW	AASW	SGLM	AALM	SGLE	AALE	SGLW	AALW
Anthraquinone	+ +	+	-	+ +	-	+ +	-	+	+	-	+ +	+ +
Saponins	+ + +	+ + +	-	-	+ +	+ + +	+ + +	+ + +	-	-	+ +	+ +
Steroids	+ +	+ +	+	+ + +	+ +	+ + +	+ +	+	-	-	+ +	+ +
Chalcones	+ +	+	+ + +	-	+	+	+	-	-	-	+	+
Phlobatannins	+	+	-	+	-	+	-	+ +	+	-	+ +	+ +
Terpenoids	+ +	+ +	+	+ + +	+ +	+ + +	+ + +	+ +	-	-	+ +	+ +
Alkaloids	+ + +	+ +	-	+ + +	+	+ +	+ + +	+ +	+	+	+ + +	+ + +
Cardiac glycosides	+	-	-	+ +	+	+ + +	+ +	+ + +	+	-	+ + +	+ +
Flavonoids	+ +	-	+ + +	+ + +	+	-	-	+ +	+ + +	+ + +	+	-
Tannins	+ + +	+ +	+ + +	+ +	+ + +	+ + +	+ + +	+ + +	+ +	+ +	+ + +	+ + +
Phenol	+	+	+ +	+ + +	+	+ + +	+	+ + +	+ + +	+ + +	+	+ + +
Polyphenols	+ +	+ +	+ + +	+ + +	+ +	+ + +	+	+ + +	+ + +	+ + +	+	+ + +
Anthocyanins	-	-	+ +	-	-	-	-	-	+	-	-	-
Quinones	-	+	+ +	-	-	+	+ +	+ + +	+ +	+	+ +	+ + +

Key: +  +  + High concentration, +  + Moderate concentration, + Low Concentrations,—Absent, *SGSM Symphonia globulifera* stem bark methanol extract, *AASM Allophylus abyssinicus* stem bark methanol extract, *SGSE Symphonia globulifera* stem bark ethyl acetate extract, *AASE Allophylus abyssinicus* stem bark ethyl acetate extract, *SGSW Symphonia globulifera* stem bark water extract, *AASW Allophylus abyssinicus* stem bark water extract, *SGLM Symphonia globulifera* leaf methanol extract, *AALM Allophylus abyssinicus* leaf methanol extract, *SGLE Symphonia globulifera* leaf ethyl acetate extract, *AALE Allophylus abyssinicus* leaf ethyl acetate extract, *SGLW Symphonia globulifera* leaf water extract, *AALW Allophylus abyssinicus* leaf water extract

Methanol has a higher extraction capacity for tannins compared to other solvents [46] and this was also proven by the observed high concentrations of tannins in methanolic extracts of leaves and stem bark of *S. globulifera* and leaves of *A. abyssinicus* (Table 1).

It was not surprising that anthraquinones were generally in low concentrations or absent in the leaves and stem bark of both *S. globulifera* and *A. abyssinicus* as they are not common within family Sapindaceae and family Clusiacea to which *S. globulifera* and *A. abyssinicus* respectively belong. Anthraquinones exist in limited groups of angiosperm families such as Fabaceae, Liliaceae, Polygonaceae, Rhamnaceae, Rubiaceae, and Scrophulariaceae [47].

The phytochemical profile of the two plants, had notable differences for example anthocyanins only occurred in the leaves and stem bark of ethyl acetate extracts of *S. globulifera* but not in *A. abyssinicus*, as shown in Table 1. This was not surprising as the composition of these secondary metabolites in plants varies from species to species [47]. The inability of methanol and water to extract the anthocyanins compounds from *S. globulifera* was due to the high polarity of these solvents which increases the polarity difference between anthocyanins and the solvent [48].

Quinones were mainly concentrated in the leaf extracts of *S. globulifera* and *A. abyssinicus* compared to the stem bark. This concentration can be attributed to their role as electron carriers during the process of photosynthesis hence their high concentration in the leaf, an organ specialized to synthesize food for the plant [49].

Thin layer chromatography (TLC) also showed a wide diversity of secondary metabolite compounds in the extracts of both *A. abyssinicus* and *S. globulifera leaves* and stem bark as determined using Hexane: Chloroform: Methanol 6:3:1 (v/v) solvent system. Generally, normal light visualization revealed a low number of compounds compared to Ultraviolent light. The increase in the number of spots was due to the fluorescence of the separated compounds when hit by Ultraviolent light hence making them more visible. Under normal light, *A. abyssinicus* leaves ethyl acetate extract showed the highest number of compounds (8 compounds) with retention factor (R_*f*_) values ranging from 0.347 to 0.972. However, *S. globulifera* leaves ethyl acetate extract had the highest number of compounds upon visualization with 245 nm (10 compounds) and 365 nm UV-light (11 compounds), with retention factor (R_f_) values ranging from 0.083 to 0.889 and 0.097 to 0.903 respectively. The wide range of retention factor values is indicative of the presence of compounds of different polarities in the same extract i.e. highly polar, highly non-polar and moderately polar. Other studies [25, 50, 51] have also shown the presence of compounds of different polarity within plant extracts. The variation in the retention factor is very important in selecting the most appropriate solvent system for further purification and isolation of the bioactive compounds within the plant extract.

TLC also revealed that the two plants shared common phytochemical compounds for example *A. abyssinicus* leaf methanol extract and *S. globulifera* leaves ethyl acetate extract both had compounds with retention factors 0.069, 0.097, 0.333, 0.903 (Table 2). Similarly, *S. globulifera* stem bark water extract and *A. abyssinicus* stem bark ethyl acetate extract both had a compound with the same retention factor (0.082) despite them belonging to different species. This may be attributed to the fact that both *S. globulifera and A. abyssinicus* are higher plants and share many metabolic pathways hence yielding the same compounds. It is also important to note that both plants were collected from the same tropical ecological and climatic conditions of Bwindi Impenetrable Forest National Park. The methanolic and water extracts of *A. abyssinicus* stem bark upon visualization under 365 nm revealed homogenous spots contrary to the many phytochemical groups reported present according to preliminary phytochemical screening (Table 1). Homogenous spots have also been reported from extracts of *Lecuas aspera* [52], *Cordia milleni* [51].

**Table 2:** Thin layer chromatography finger print of the stem bark and leaves’ extracts of *Symphonia globulifera* and *Allophylus abyssinicus*

**Extract Number (Code)**	**Under Normal light**			**Under 245 nm**		**Under 365 nm**	
	**Spots**	**Spot Color**	**Retention factor (R**_**f**_**)**	**Spots**	**Retention factor (R**_**f**_**)**	**Spots**	**Retention factor (R**_**f**_**)**
**1 (SGLM)**	1	Yellowish Brown	0.085	2	0.085	2	0.056
					0.887		0.099
**4 (AALM)**	5	Light Green	0.389	5	0.097	8	0.069
		Yellowish Green	0.431		0.403		0.097
					0.431		0.167
		Green	0.458		0.472		0.333
		Yellowish Green	0.847		0.861		0.417
							0.472
		Light Green	0.889				0.875
							0.903
**2 (SGLE)**	7	Yellow Brown	0.306	10	0.083	11	0.069
					0.222		0.097
		Light Green	0.375		0.333		0.208
		Yellowish Green	0.417		0.361		0.333
					0.417		0.389
		Green	0.444		0.444		0.444
		Light Yellow	0.667		0.569		0.514
		Yellowish Green	0.875		0.583		0.583
					0.861		0.681
		Green	0.889		0.889		0.889
							0.903
**5 (AALE)**	8	Yellow Brown	0.347	8	0.333	8	0.056
		Green	0.375		0.361		0.083
		Dark Green	0.417		0.417		0.361
		Yellow	0.667		0.444		0.403
		Light Orange	0.778		0.583		0.431
		Yellow green	0.861		0.861		0.639
		Dark green	0.903		0.903		0.861
		Yellow	0.972		0.958		0.903
**3 (SGLW)**						4	0.083
							0.139
							0.292
							0.486
**6 (AALW)**				1	0.083	2	0.056
							0.083
**1' SGSM)**				1	0.083	4	0.069
							0.111
							0.417
							0.556
**4' AASM)**						1	0.083
**2' (SGSE)**	4	Orange brown	0.384	3	0.055	9	0.055
		Orange brown	0.493		0.466		0.096
		Yellow Green	0.671		0.630		0.219
		Light Yellow	0.890				0.397
							0.466
							0.630
							0.699
							0.822
							0.890
**5' (AASE)**	1	Light Green	0.890	3	0.055	4	0.082
					0.438		0.425
					0.877		0.548
							0.781
**3' SGSW)**						2	0.082
							0.438
**6' AASW)**				1	0.055	1	0.096

Key: *SGLW Symphonia globulifera* leaf water extract, *SGLE Symphonia globulifera* leaf ethyl acetate extract, *SGLM Symphonia globulifera* leaf methanol extract, *SGSW Symphonia globulifera* stem bark water extract, *SGSE Symphonia globulifera* stem bark ethyl acetate extract, *SGSM Symphonia globulifera* stem bark methanol extract, *AALW Allophylus abyssinicus* leaf water extract, *AALM Allophylus abyssinicus* leaf methanol extract, AALE *Allophylus abyssinicus* leaf ethyl acetate extract, *AASW Allophylus abyssinicus* stem bark water extract, *AASM Allophylus abyssinicus* stem bark methanol extract, *AASE Allophylus abyssinicus* stem bark ethyl acetate extract

Upon spraying the TLC plate with concentrated sulphuric acid and heating the TLC plate, it confirmed the presence of phenolic compounds in all plant extracts. This was agreeing with the preliminary phytochemical screaming which showed the presence of phenols in all extracts and phenolic compounds like flavonoids and tannins among others as in Table 1. *Allophylus abyssinicus* leaves ethyl acetate extract had the highest number of phenolic compounds (11 compounds) with Rf values ranging from 0.083 to 0.958 followed by *S. globulifera* stem bark ethyl acetate (9 compounds) with Rf values of 0.055 to 0.959. Homogenous spots were observed for *S. globulifera* stem bark water extract, *A. abyssinicus* stem bark water extract, *A. abyssinicus* stem bark methanol extract, *S. globulifera* stem bark methanol extract and *S. globulifera* leaves methanol extract. The concentrations and composition of phenolic metabolites in plants are influenced by many factors, including soil and climatic conditions [53]. It has been reported that highly polar solvents like water and methanol are more effective in the extraction of a large amount of phenolic contents plants [54], however, in this study we note the high ability of ethyl acetate, a moderately polar solvent in extracting a high number of phenolic compounds with different retention factor values compared to the highly polar methanol and water.

The various identified phytochemical compounds have been recorded to have antimicrobial activity [55–59]. It was therefore expected that all extracts of *S. globulifera* and *A. abyssinicus* should have antimicrobial activity.

### Antimicrobial activity of *Symphonia globulifera* and *Allophyllus abyssinicus* plant extracts and extract combinations

#### Antimicrobial Zones of Inhibition

Methanol and water extracts of *S. globulifera* and *A. abyssinicus* and selected combinations showed inhibition against *S. aureus*, *E. coli* and *P. aeruginosa* as summarised in Tables 3, 4 and 5 respectively. The antimicrobial activity of these extracts is attributed to the presence of bioactive compounds like tannins, flavonoids and alkaloids among others. Plant secondary metabolites have therapeutic action against pathogens and parasites [60, 61]. *Allophylus abyssinicus* leaf water extract (AALW) had the highest mean zone of inhibition against *S. aureus* at concentrations of 1000 mg/ml, 500 mg/ml, 250 mg/ ml, 125 mg/ml and 62.5 mg/ml (31.25 ± 0.50 mm, 30.00 ± 0.00 mm, 27.25 ± 0.50 mm, 24.00 ± 1.15 mm, 19.00 ± 0.00 mm respectively), followed by *A. abyssinicus* stem bark water extract (AASW) while extract combination (*S. globulifera* leaf water extract and *A. abyssinicus* leaf water extract) had the lowest mean zone of inhibition at each of the same concentrations against *S. aureus.* The high activity of *A. abyssinicus* leaf water extract compared to other extracts could be due to the presence of quinones, alkaloids, polyphenols, and phenols in high concentrations in this extract and the other phytochemical groups in moderate concentrations. The high activity of water extracts observed in this study also justifies the usual practice of traditional medicine practitioners of preparing herbal medicine using water as the main solvent. Quinones exert interesting antibacterial activity against *S. aureus* by donating free radicals which form irreversible complexes with amino acids in proteins hence easily attacking the cell walls and membrane enzymes of gram-positive bacteria like *S. aureus* to inactivation [62, 63]*.* Crude extracts from other *Allophylus* species like *A. cobbe* and *A. serratus* showed the maximum zone of inhibition in the range of 20–23 mm, comparatively less than standard antibiotic Cefotaxime against *S. aureus* [13]. Oliveira and collegues [64] also demonstrated larger zone of inhibition of aqueous extracts of *S. globulifera* against *S. aureus* relatable to those shown in this study (Table 3). The significantly higher zones of inhibition of the aqueous leaf extracts of both *A. abyssinicus* and *S. globulifera* than the stem bark extracts respectively against *S. aureus* is highly advantageous in sustainable utilization and conservation of the two plants. The collection of leaves for herbal medicine causes less damage to plants compared to the harvesting of the stem bark because the stem bark takes long periods to heal. Increasing demand and destructive harvesting of stem bark rather than leaves has led to the depletion of several valuable medicinal trees and threatens the continued availability of medicinal products from these trees [22].

**Table 3:** Mean ± SD Zone of Inhibition of *Symphonia globulifera* and *Allophylus abyssinicus* extracts and combinations against *Staphylococcus aureus*

Extract	Mean ± SD Zone of Inhibition (mm) of *Staphylococcus aureus*										
	**1000** **mg/ml**	**500** **mg/ml**	**250** **mg/ml**	**125** **mg/ml**	**62.5** **mg/ml**	**31.25** **mg/ml**	**15.62** **mg/ml**	**7.81** **mg/ml**	**3.90** **mg/ml**	**1.95** **mg/ml**	**Ciproflo** **xacin**
AALW	31.25 ± 0.50^a^	30.00 ± 0.00^a^	27.25 ± 0.50^a^	24.00 ± 1.15^a^	19.00 ± 0^a^	7.25 ± 0.50^ cd^	3.75 ± 0.50^ cd^	0	0	0	20.50 ± 3.10
AALM	19.00 ± 0.00^de^	14.50 ± 1.00^ghi^	12.25 ± 0.96^ fg^	11.00 ± 0^df^	8.50 ± 0.56^e^	7.00 ± 0.00^d^	3.00 ± 0.82^de^	0.75 ± 0.96^ g^	0	0	24.00 ± 0.81
AASW	26.75 ± 1.50^b^	24.50 ± 0.58^b^	21.75 ± 0.50^b^	19.75 ± 1.70^b^	15.00 ± 0.00^b^	9.75 ± 0.50^a^	7.00 ± 0.82^a^	3.25 ± 0.96^ab^	0	0	20.25 ± 0.50
AASM	17.75 ± 0.96^eg^	15.00 ± 0.82^fh^	12.00 ± 0^ fg^	10.75 ± 0.96^ef^	8.75 ± 0.96^e^	7.25 ± 0.50^ cd^	4.75 ± 0.96^b^	1.00 ± 1.41^ fg^	0	0	27.00 ± 0.82
SGLW	22.75 ± 0.96^c^	17.25 ± 0.50^c^	14.75 ± 0.50^d^	12.00 ± 0^d^	10.50 ± 0.58^d^	8.00 ± 0^bc^	6.25 ± 0.96^a^	2.75 ± 0.50^bd^	0	0	32.50 ± 1.00
SGLM	20.25 ± 0.50^d^	16.00 ± 0.00^def^	12.00 ± 0^ fg^	10.00 ± 0.82^ fg^	5.00 ± 0.00^ g^	3.00 ± 0.82^ g^	0	0	0	0	27.00 ± 0.00
SGSW	18.25 ± 0.96^e^	15.25 ± 0.96^ fg^	12.75 ± 0.96^ef^	10.00 ± 1.41^ fg^	7.00 ± 0.00^f^	5.25 ± 0.50^f^	3.50 ± 0.58^ce^	1.25 ± 1.26^ fg^	0	0	24.00 ± 0
SGSM	18.00 ± 0.82^ef^	16.50 ± 0.58^ce^	12.50 ± 0.58^eg^	10.00 ± 0^ fg^	7.00 ± 0.82^f^	3.75 ± 0.96^ g^	3.00 ± 0.82^de^	0	0	0	24.75 ± 0.96
SGLW + AALW	15.00 ± 0.82^i^	12.25 ± 0.96^ k^	10.25 ± 0.96^ h^	6.75 ± 0.50	5.00 ± 0.00^ g^	3.00 ± 0.82^ g^	0	0	0	0	18.25 ± 0.96
SGLW + SGLM	18.25 ± 0.96^e^	14.75 ± 0.96^gh^	12.75 ± 0.50^ef^	9.25 ± 0.50^gh^	6.75 ± 0.50^f^	6.00 ± 0^ef^	4.25 ± 0.96^bc^	1.75 ± 0.50^ deg^	0	0	22.25 ± 1.71
AASW + AALW	15.50 ± 1.29^hi^	13.00 ± 0.82^ k^	12.00 ± 0.00^ fg^	11.25 ± 0.96^de^	10.00 ± 8.62^d^	8.75 ± 0.50^b^	7.00 ± 0.00^a^	4.25 ± 0.50^a^	1.50 ± 0.57^ab^	0	22.75 ± 0.97
SGLM + AASW	15.25 ± 1.50^i^	12.75 ± 0.96^jk^	10.25 ± 0.50^ h^	8.25 ± 0.50^ h^	7.00 ± 0.00^f^	5.75 ± 0.96^f^	3.50 ± 0.58^ce^	2.00 ± 0.82^cdef^	0.75 ± 0.50^c^	0	25.75 ± 1.26
SGLW + AASW	17.75 ± 0.96^eg^	16.75 ± 0.50^ cd^	15.75 ± 0.50^c^	14.00 ± 0^c^	11.50 ± 1.29^c^	7.25 ± 0.96^ cd^	4.25 ± 0.50^bc^	2.50 ± 0.58^be^	1.00 ± 0.82^bc^	0	22.00 ± 0
AALW + SGLM	16.75 ± 0.50^fgh^	13.50 ± 1.29^ij^	13.00 ± 0^e^	11.25 ± 0.96^de^	9.75 ± 0.50^d^	6.75 ± 0.50^de^	5.00 ± 0.82^b^	3.00 ± 0.82^bc^	2.00 ± 0.82^a^	0	23.50 ± 1.00

^a^^−^^j^*In columns mean values sharing a superscript letter are not significantly different (p* > *0.05) as based on the LSD multiple Comparison test*

*AALW* *Allophylus* abyssinicus *leaf water extract, AALM* *Allophylus abyssinicus* *leaf methanol extract, AASW* *Allophylus abyssinicus* *stem bark water extract, AASM* *Allophylus abyssinicus* *stem bark methanol extract, SGLW* *Symphonia globulifera* *leaf water extract, SGLM* *Symphonia globulifera* *leaf methanol extract, SGSW* *Symphonia globulifera stem bark water extract, SGSM* *Symphonia globulifera* *stem bark methanol extract*

**Table 4:** Mean ± SD Zone of Inhibition of *Symphonia globulifera* and *Allophylus abyssinicus* extracts and combinations against *Escherichia coli*

Extract	Mean ± SD Zone of Inhibition (mm) of *Escherichia coli*										
	**1000** **mg/ml**	**500** **mg/ml**	**250** **mg/ml**	**125** **mg/ml**	**62.5** **mg/ml**	**31.25** **mg/ml**	**15.62** **mg/ml**	**7.81** **mg/ml**	**3.90** **mg/ml**	**1.95 mg/ml**	**Cipro** **floxacin**
AALW	26.75 ± 0.96^a^	22.00 ± 0^a^	20.00 ± 0^a^	15.25 ± 0.96^a^	10.25 ± 0.50^b^	8.00 ± 0.00^b^	4.50 ± 0.58^a^	1.00 ± 0.82^a^	0	0	37.00 ± 0
AALM	20.25 ± 0.50^d^	13.75 ± 0.96^e^	12.00 ± 0^d^	9.00 ± 0^f^	8.50 ± 0.58^c^	7.00 ± 0.82^b^	4.00 ± 0^ab^	1.25 ± 0.50^a^	0	0	36.50 ± 1.00
AASW	22.00 ± 0^bc^	17.00 ± 0^d^	11.25 ± 0.96^de^	6.75 ± 0.50^ g^	4.00 ± 0^f^	1.75 ± 0.50	0	0	0	0	37.00 ± 0.00
AASM	13.00 ± 0.82^ h^	8.75 ± 0.96^ h^	6.75 ± 0.50^ g^	3.50 ± 0.58^ h^	2.25 ± 0.50^ g^	0	0	0	0	0	32.75 ± 0.96
SGLW	26.00 ± 0.82^a^	20.25 ± 0.50^b^	16.25 ± 0.50^b^	14.75 ± 0.50^a^	12.00 ± 0^a^	9.25 ± 0.96^a^	4.50 ± 0.58^a^	0	0	0	34.00 ± 2.45
SGLM	22.75 ± 0.96^b^	19.00 ± 0.82^c^	8.00 ± 1.83^ g^	0	0	0	0	0	0	0	32.00 ± 0.82
SGSW	15.75 ± 0.96^ef^	9.75 ± 0.96^ g^	4.50 ± 1.29^i^	2.25 ± 0.50^i^	0	0	0	0	0	0	32.50 ± 1.0
SGSM	13.25 ± 0.50^gh^	6.75 ± 0.50^i^	4.25 ± 0.50^i^	0	0	0	0	0	0	0	32.75 ± 0.96
SGLW + AALW	16.25 ± 0.96^e^	13.25 ± 0.50^e^	12.00 ± 0^d^	10.25 ± 0.50^ cd^	7.50 ± 1.00^d^	4.25 ± 2.50^def^	0	0	0	0	34.00 ± 2.45
SGLW + SGLM	20.75 ± 1.50^ cd^	16.75 ± 0.96^d^	13.75 ± 1.26^c^	9.25 ± 0.50^ef^	5.75 ± 1.50^e^	0	0	0	0	0	38.50 ± 2.38
AASW + AALW	14.50 ± 0.58^ fg^	12.25 ± 0.50^f^	11.00 ± 0.82^df^	10.00 ± 0.82^de^	7.00 ± 0.82^d^	5.00 ± 0.82^ce^	4.00 ± 0^ab^	1.25 ± 0.96^a^	0	0	35.00 ± 2.45
SGLM + AASW	20.00 ± 0.82^d^	18.50 ± 0.58^c^	14.75 ± 0.96^c^	11.25 ± 0.50^b^	7.75 ± 0.50^ cd^	5.50 ± 0.58^c^	3.50 ± 0.58^b^	0	0	0	32.75 ± 0.96
SGLW + AASW	20.25 ± 1.26^d^	18.25 ± 0.50^c^	14.00 ± 0^c^	11.00 ± 0.82^bc^	8.50 ± 0.58^c^	5.25 ± 0.96^ cd^	2.75 ± 1.26^c^	0	0	0	36.00 ± 1.83
AALW + SGLM	16.50 ± 1.29^e^	13.00 ± 0.82^ef^	10.50 ± 1.00^ef^	7.25 ± 0.50^ g^	5.25 ± 0.96^e^	3.50 ± 0.58^f^	1.75 ± 0.50	0	0	0	34.50 ± 3.00

^a^^−^^j^ In columns mean values sharing a superscript letter are not significantly different (*p* > 0.05) as based on the LSD multiple Comparison test

*AALW Allophylus abyssinicus* leaf water extract, *AALM Allophylus abyssinicus* leaf methanol extract, *AASW Allophylus abyssinicus* stem bark water extract, *AASM Allophylus abyssinicus* stem bark methanol extract, *SGLW Symphonia globulifera* leaf water extract, *SGLM Symphonia globulifera* leaf methanol extract, *SGSW Symphonia globulifera* stem bark water extract, *SGSM Symphonia globulifera* stem bark methanol extract

**Table 5:** Mean ± SD Zone of Inhibition of *Symphonia globulifera* and *Allophylus abyssinicus* extracts and combinations *against Pseudomonas aeruginosa*

Extract	Mean ± SD Zone of Inhibition (mm) of *Pseudomonas aeruginosa*										
	**1000** **mg/ml**	**500** **mg/ml**	**250** **mg/ml**	**125** **mg/ml**	**62.5** **mg/ml**	**31.25** **mg/ml**	**15.62** **mg/ml**	**7.81** **mg/ml**	**3.90** **mg/ml**	**1.95 mg/ml**	**Cipro** **floxacin**
AALW	24.75 ± 1.89^ac^	15.00 ± 2.16^efg^	10.75 ± 0.50^d^	6.75 ± 0.50^ g^	4.25 ± 0.50	2.50 ± 0.58^d^	0.75 ± 0.50^d^	0	0	0	21.75 ± 1.26
AALM	19.75 ± 0.96^ef^	15.75 ± 0.50^df^	10.75 ± 0.50^d^	4.25 ± 1.50^hi^	3.00 ± 0.00	1.00 ± 0.82^e^	0	0	0	0	16.75 ± 0.50
AASW	20.25 ± 0.96^e^	16.50 ± 1.00^d^	14.00 ± 0.82^b^	12.00 ± 0^b^	9.00 ± 0.82^c^	5.50 ± 1.29^b^	2.25 ± 0.50^c^	0.75 ± 0.50^b^	0	0	21.00 ± 0.82
AASM	19.50 ± 1.00^eg^	15.50 ± 0.58^dg^	13.00 ± 0^bc^	10.75 ± 0.96^ cd^	8.75 ± 0.50^c^	5.00 ± 0.82^b^	4.00 ± 1.63^a^	3.25 ± 0.96^a^	0.50 ± 0.00^a^	0	21.75 ± 0.96
SGLW	24.00 ± 1.41^bcd^	21.00 ± 0.82^a^	16.75 ± 0.96^a^	14.25 ± 0.96^a^	11.25 ± 0.50^a^	3.50 ± 0.58^ cd^	0	0	0	0	17.00 ± 0.82
SGLM	16.25 ± 1.26^ h^	11.00 ± 0.82^ h^	6.50 ± 0.58^e^	4.00 ± 0.82^hj^	2.00 ± 0.82^f^	1.25 ± 0.50^e^	0	0	0	0	20.75 ± 0.96
SGSW	16.25 ± 0.96^ h^	13.25 ± 0.50	7.00 ± 0.82^e^	3.25 ± 0.50^ij^	0	0	0	0	0	0	17.50 ± 1.29
SGSM	16.00 ± 0.82^ h^	10.75 ± 0.96^ h^	7.00 ± 0.82^e^	4.50 ± 0.58^ h^	1.75 ± 0.96^f^	0.75 ± 0.50^e^	0	0	0	0	20.75 ± 0.96
SGLW + AALW	25.75 ± 1.50^a^	20.75 ± 0.96^ab^	16.25 ± 0.50^a^	12.75 ± 0.96^b^	10.25 ± 0.50^b^	7.00 ± 1.41^a^	3.75 ± 0.50^ab^	1.50 ± 0.58^b^	0	0	19.00 ± 0.82
SGLW + SGLM	23.00 ± 0.82^d^	15.75 ± 0.96^df^	12.25 ± 0.50^c^	9.25 ± 0.50^e^	6.25 ± 0.96^de^	3.50 ± 0.58^ cd^	0	0	0	0	17.75 ± 0.50
AASW + AALW	20.25 ± 0.96^e^	16.00 ± 0^de^	13.75 ± 0.50^b^	11.75 ± 0.50^bc^	9.00 ± 0.82^c^	5.00 ± 1.16^b^	3.00 ± 0^bc^	1.50 ± 0.56^b^	0.50 ± 0.58^a^	0	21.00 ± 0.82
SGLM + AASW	23.00 ± 0.00^d^	19.25 ± 0.96^c^	13.75 ± 1.50^b^	10.00 ± 0.00^de^	7.00 ± 0.82^d^	3.75 ± 0.50^c^	1.25 ± 0.96^d^	0	0	0	20.75 ± 0.96
SGLW + AASW	18.25 ± 0.50^ fg^	15.50 ± 1.00^dg^	12.00 ± 0.82^c^	8.00 ± 0.82^f^	5.75 ± 0.50^e^	3.50 ± 0.58^ cd^	1.25 ± 0.96^d^	0	0	0	20.25 ± 0.50
AALW + SGLM	25.25 ± 2.22^ab^	19.50 ± 1.00^bc^	16.75 ± 0.96^a^	12.25 ± 0.96^b^	10.75 ± 0.50^ab^	0	0	0	0	0	21.25 ± 0.96

^a^^−^^j^ In columns mean values sharing a superscript letter are not significantly different (*p* > 0.05) as based on the LSD multiple Comparison test

AALW *Allophylus abyssinicus* *leaf water extract,* AALM *Allophylus abyssinicus* *leaf methanol extract,* AASW *Allophylus abyssinicus* *stem bark water extract,* AASM *Allophylus abyssinicus* *stem bark methanol extract,* SGLW *Symphonia globulifera* *leaf water extract,* SGLM *Symphonia globulifera* *leaf methanol extract,* SGSW *Symphonia globulifera* *stem bark water extract,* SGSM *Symphonia globulifera* *stem bark methanol extract*

The methanolic, water extracts and selected combinations of *S. globulifera* and *A. abyssinicus* also inhibited the growth of *E. coli* as shown in Table 4*.* At concentrations of 1000 to 250 mg/ml *A. abyssinicus* leaf water extract showed the largest zone of inhibition against *E. coli* (26.75 ± 0.96 mm, 22.00 ± 0 mm and 20.00 ± 0 mm). This shows the presence of bioactive compounds with activity against gram-negative bacteria like *E. coli*. However, AALW showed a smaller zone of inhibition against *E. coli* compared to *S. aureus*. This reduced activity against gram-negative *E. coli* was not surprising as plant extracts are usually more active against gram-positive bacteria than gram-negative bacteria due to structural differences in the cell wall. Gram-negative bacteria possess an outer membrane with a hydrophilic surface that functions as a permeability barrier against many natural compounds [65].

This study presents novel evidence of growth inhibition of *P. aeruginosa* by *S. globulifera* and *A. abyssinicus.* Extract combinations (*S. globulifera* leaf water extract and *A. abyssinicus* leaf water extract, *A. abyssinicus* leaf water extract and *S. globulifera* leaf methanol extract) generally showed the largest zones of inhibition against *P. aeruginosa* compared to the individual single extracts (Table 5). *Pseudomonas aeruginosa* is highly resistant and easily acclimates to the new environment, which probably explains its minimal susceptibility to individual plant extracts. *P. aeruginosa* has a large genome size ranging between 5.5–7 Mbp which enables it to encode for a large proportion of regulatory enzymes important for metabolism, transportation and efflux of organic compounds hence metabolic versatility and high adaptability potential to environmental stresses [66]. Among the individual extracts, at 1000 mg/ml, *A. abyssinicus* leaf water extract showed the highest zone of inhibition (24.75 ± 1.89 mm) against *P. aeruginosa* but this was not significantly different from the mean zone of inhibition by *S. globulifera* leaf water extract (24.00 ± 1.41 mm), this trend may be attributed to common phytochemical compounds like tannins and alkaloids in higher concentrations in both extracts. Tannins have a highly selective action for *P. aeruginosa* with a bacteriostatic mode of action by impairing its adhesion onto surfaces [67]. Alkaloids from leaves of the *Callistemon citrinus* could inhibit ATP-dependent transport efflux pumps in *P. aeruginosa* [57].

The plant extracts of *A. abyssinicus* and *S. globulifera* showed small zones of inhibition at high concentrations (1000 mg/ml and 500 mg/ml) against the growth of *C. albicans* as shown in Table 6. At 1000 mg/ ml of extracts, SGSM had the highest zone of inhibition (13.00 ± 1.41 mm) against *C. albicans* and AASW had the lowest mean zone of inhibition (5.75 ± 6.28 mm) against *C. albicans.* This minimal anti-*Candida albicans* activity from the extracts of *A. abyssinicus* and *S. globulifera* may be caused by low and moderate concentrations of anthraquinones, chalcones and anthocyanins within the extracts. Anthraquinones, chalcones and anthocyanins possess antifungal activity properties [68]. All four methanolic extracts of the two plants inhibited the growth of *C. albicans* unlike the water extracts, this is indicative of the high capacity of methanol to extract antifungal phytochemicals compared to water. Methanolic extracts of plants like Lamiaceae species and *Papaver rhoeas* L. have shown strong antifungal activity against *Candida* species*, Fusarium* species and *Aspergillus* species [69, 70]*.*

**Table 6:** Mean ± SD Zone of Inhibition of *Symphonia globulifera* and *Allophylus abyssinicus* Extracts and Combinations against Candida albicans

**Extract**	**Mean ± SD Diametric Zone of Inhibition (mm) of *Candida albicans***										**Fluconazole**
	**1000** **mg/ml**	**500** **mg/ml**	**250** **mg/ml**	**125** **mg/ml**	**62.5** **mg/ml**	**31.25** **mg/ml**	**15.62** **mg/ml**	**7.81 mg/ml**	**3.90** **mg/ml**	**1.95** **mg/ml**	
AALW	0	0	0	0	0	0	0	0	0	0	32.75 ± 0.96
AALM	11.25 ± 0.96^ac^	3.25 ± 0.96^a^	0	0	0	0	0	0	0	0	32.00 ± 0.00
AASW	5.75 ± 6.28^e^	4.00 ± 4.28^a^	0	0	0	0	0	0	0	0	33.00 ± 0.83
AASM	12.00 ± 0.82^ab^	2.75 ± 1.50^a^	0	0	0	0	0	0	0	0	33.50 ± 1.00
SGLW	0	0	0	0	0	0	0	0	0	0	35.00 ± 2.45
SGLM	7.25 ± 0.50^de^	0	0	0	0	0	0	0	0	0	45.00 ± 0
SGSW	0	0	0	0	0	0	0	0	0	0	37.75 ± 0.96
SGSM	13.00 ± 1.41^a^	0	0	0	0	0	0	0	0	0	43.25 ± 2.50
SGLW + AALW	0	0	0	0	0	0	0	0	0	0	33.25 ± 0.96
SGLW + SGLM	0	0	0	0	0	0	0	0	0	0	33.00 ± 0.82
AASW + AALW	0	0	0	0	0	0	0	0	0	0	33.25 ± 0.96
SGLM + AASW	0	0	0	0	0	0	0	0	0	0	33.25 ± 1.26
SGLW + AASW	9.00 ± 0.82^bcd^	5.25 ± 0.96^a^	0	0	0	0	0	0	0	0	33.50 ± 0.58
AALW + SGLM	9.00 ± 0.82^bcd^	5.25 ± 096^a^	0	0	0	0	0	0	0	0	34.00 ± 0.00

^a^^−^^j^ In columns mean values sharing a superscript letter are not significantly different (p > 0.05) as based on the LSD multiple Comparison test

*AALW Allophylus abyssinicus* leaf water extract, *AALM Allophylus abyssinicus* leaf methanol extract, *AASW Allophylus abyssinicus* stem bark water extract, *AASM Allophylus abyssinicus* stem bark methanol extract, *SGLW Symphonia globulifera* leaf water extract, *SGLM Symphonia globulifera* leaf methanol extract, *SGSW Symphonia globulifera* stem bark water extract, *SGSM Symphonia globulifera* stem bark methanol extract

#### Antimicrobial Minimum Inhibitory concentration 

The MICs’ of extracts against *S. aureus*, *E. coli* and *P. aeruginosa* ranged from 7.81 mg/ml to 31.25 mg/ml, 7.81 mg/ml to 250 mg/ml and 3.90 mg/ml to 125 mg/ml respectively as shown in Table 7 . Generally, extract combinations showed the lowest MICs values against *S. aureus*, *E. coli* and *P. aeruginosa* ranging from 3.90 mg/ml to 31.25 mg/ml, 7.81 mg/ml to 31.25 mg/ml and 3.90 mg/ml to 6.25 mg/ml respectively (Table 7) indicative of possible synergistic activity between the extracts. Extract combinations (AASW and AALW, SGLM and AASW, SGLW and AASW, AALW and SGLM) had a homogenous, lowest MIC (3.90 mg/ml) against *S. aureus*. Similarly, the combination of *A. abyssinicus* stem bark water extract and *A. abyssinicus* leaves water extract (AASW & AALW) had the lowest MIC (7.81 mg/ml and 3.90 mg/ml) against both *E. coli* and *P. aeruginosa* respectively. The higher anti-bacterial activity of extract combinations than single extracts justifies the practice of using more than one plant for the development of herbal therapeutics by herbal medicine practitioners. The therapeutic value of synergistic interactions is relied upon by herbal healing systems to obtain enhanced efficacy against ailments [41]. The MIC of the methanolic extracts of *S. globulifera* leaves and stem bark (15.62 mg/ml) against *S. aureus* in the current study were relatively higher than that of the methanol extract of the seeds of *S. globulifera* (MIC = 0.3 mg/ml) as reported by Lenta and collegues [71]. This low antimicrobial activity of the *S. globulifera* extracts of the current study compared to Lenta and collegues [71] can be due to differences in phytochemical composition as a result of differences in soil types and climate between the Bwindi Impenetrable forest, Uganda and the North-west province of Cameroon. *Symphonia globulifera* has a variety of isolated compounds with good anti- *Staphylococcus aureus* activity compared to the crude methanol extracts in the current study, for example, Biflavonoïd GB2, Manniflavanone GB3, Globulixanthone F, have lower MICs’ 8.50 µg/ml, 8.50 µg/ml and 4.50 µg/ml respectively against *S. aureus* [72]. However, the mode of action whether bacteriostatic or bactericidal of these compounds remains unknown.

**Table 7:** The minimum inhibitory concentration (MIC) of singular *Symphonia globulifera* and *Allophylus abyssinicus* extracts and their combinations

**Extract**	**Minimum Inhibitory Concentration (mg/ml)**			
	***Staphylococcus*** ***aureus*** **ATCC 25,923**	***Escherichia coli*** **ATCC25922**	***Pseudomonas*** ***aeruginosa*** **ATCC 27,853**	***Candida*** ***albicans* ATCC 10,231**
AALW	15.62	7.81	7.81	N/A
AALM	7.81	7.81	31.25	500.00
AASW	7.81	31.25	7.81	500.00
AASM	7.81	62.50	3.90	500.00
SGLW	7.81	15.62	31.25	N/A
SGLM	31.25	250.00	31.25	1000.00
SGSM	15.62	250.00	31.25	1000.00
SGSW	7.81	125.00	125.0	N/A
SGLW + AALW	31.25	31.25	7.81	N/A
SGLW + SGLM	7.81	15.62	31.25	500.00
AASW + AALW	3.90	7.81	3.90	N/A
SGLM + AASW	3.90	15.62	15.62	N/A
SGLW + AASW	3.90	15.62	15.62	1000.00
AALW + SGLM	3.90	15.62	62.50	500.00
Ciprofloxacin (µg/ml)	0.26	0.02	0.02	N/A
Fluconazole (µg/ml)	N/A	N/A	N/A	4.00

Key: *N/A* No antimicrobial activity, *AALW Allophylus abyssinicus* leaf water extract, *AALM Allophylus abyssinicus* leaf methanol extract, *AASW Allophylus abyssinicus* stem bark water extract, *AASM Allophylus abyssinicus* stem bark methanol extract, *SGLW Symphonia globulifera* leaf water extract, *SGLM Symphonia globulifera* leaf methanol extract, *SGSM Symphonia globulifera* stem bark methanol extract, *SGSW Symphonia globulifera* stem bark water extract

All extracts and combinations except AALM had lower MIC against *S. aureus* than *E. coli* indicating a stronger antimicrobial activity against *S. aureus* than *E. coli* possibly due to the presence of an outer membrane in the cell wall of *E. coli*, that acts as a permeability barrier preventing the penetration of the bioactive compound into *E. coli* cells. Lower MIC values of plant extracts against *S. aureus* compared to *E. coli* have also been reported [73, 74].

Surprisingly, AALM had the same MIC (7.81 mg/ml) for both *E. coli* and *S. aureus,* this is evocative of broad-spectrum antibiotic compounds with activity against both gram-positive and gram-negative bacteria.

AASM and extract combination (AASW and AALW) had the highest antimicrobial activity (MIC = 3.90 mg/ml) against *P. aeruginosa* as shown in Table 7. AASM and AASW had a high concentration of saponins which have bactericidal activity and inhibit the formation of biofilms, which exposes *P. aeruginosa* cells to the activity of other bioactive compounds [75]. Combinations SGLM and AASW, SGLW and AASW had the same MIC values for *E. coli* and *P. aeruginosa* probably because both are gram-negative bacteria with many common structures as drug targets.

The extracts and combinations had high MIC values (1000 mg/ml and 500 mg/ml) (Table 7) against *C. albicans* interpretable as a low antifungal activity just as indicated by the zone of inhibitions (Table 6). This low antifungal activity could be attributed to the usage of brain heart infusion broth instead of RPMI 1640 medium supplemented with morpholinepropanesulfonic acid (MOPS) as recommended by the Clinical and Laboratory Standards Institute (CLSI) guidelines for antifungal activity testing. A similar study comparing the antifungal activity of fungal agents (hydroxy-itraconazole and itraconazole) against *Candida albicans* growing in different media including brain heart infusion (BHI), showed that the media significantly influences the determination of MIC end points in that MIC end points of both drugs could not be determined in brain heart infusion [76]. Furthermore, this low antifungal activity is probably due to limited concentrations of antifungal compounds like anthraquinones and anthocyanins that have been reported as antifungal [77–79] with in the extracts. AALW had no anti-fungal activity however its combination with SGLM showed activity (MIC = 500 mg/ml) compared to that of SGLM (MIC = 1000 mg/ml) as a result of synergistic interaction between the two species. It has been earlier reported that plant extracts with no antimicrobial activity when mixed with antimicrobial active compounds, may induce a much stronger antimicrobial activity [80]. For example, chlorhexidine’s zone of inhibition against *C. albicans* was 30.3–19.3 mm, but in combination with the ethyl acetate extract (100 mg/mL) of *Tanacetum vulgare*, there was an increase in the inhibition (32.7–30 mm), indicating synergistic effect [81].

#### Minimum Bactericidal Concentration (MBC)

The MBC of the extracts against *S. aureus* ATCC 2592*, E. coli* ATCC 25,922 and *P. aeruginosa* ATCC 27,853 ranged between 15.62 mg/ml to 500 mg/ml, 250 mg/ml to 500 mg/ml and 250 mg/ml to 500 mg/ml respectively as shown in Table 8. These results in Table 8, indicate that the plant extracts had variable bactericidal activity. *Symphonia globulifera* leaf water extract had the lowest minimum bactericidal concentration (15.625 g/ml) against *S. aureus.*

**Table 8:** The minimum bactericidal and fungicidal concentration of *Allophylus abyssinicus* and *Symphonia globulifera* extracts

**Extract**	**MBC (mg/ml)**			**MFC (mg/ml)**
	***Staphylococcus*** ***aureus*** ***ATCC 25923***	***Escherichia*** ***coli*** **ATCC 25922**	***Pseudomonas*** ***aeruginosa*** ***ATCC 27853***	***Candida*** ***Albicans*** ***ATCC 10231***
AALW	31.25	250.00	500.00	N/A
AALM	62.50	250.00	250	^a^
SGLW	15.62	500.00	250	N/A
SGLM	125.00	500.00	250	^a^
AASW	31.25	^a^	250	N/A
SGSM	250.00	250.00	250	^a^
SGSW	500.00	500.00	250	N/A
AASM	250.00	250.00	^a^	^a^

Key: *N/A* No antifungal Activity, *AALW Allophylus abyssinicus* leaf water extract, *AALM Allophylus abyssinicus* leaf methanol extract, *SGLW Symphonia globulifera* leaf water extract, *SGLM Symphonia globulifera* leaf methanol extract, *AASW Allophylus abyssinicus* stem bark water extract, *SGSM Symphonia globulifera* stem bark methanol extract, *SGSW Symphonia globulifera* stem bark water extract, *AASM Allophylus abyssinicus* stem bark methanol extract

^a^Bacteriostatic or fungistatic extract

There was less variation in the MBC values of the extracts against *E. coli. Allophylus abyssinicu*s leaves water extract, *A. abyssinicus* leaves methanol extract, *A. abyssinicus* stem bark methanol extract and *S. globulifera* stem bark methanol extract showed the lowest but same MBC (250 mg/ml) against *E. coli.* Similarly, five of the extracts (AALM, SGLW, SGLM, AASW, SGSM, SGSW) had the lowest MBC (250 mg/ml*)* against *P. aeruginosa.* The water and methanolic stem bark extracts of *A. abyssinicus* had a bacteriostatic activity against *E. coli* and *P. aeruginosa* respectively. The mode of action of the plant extracts that leads to the death of the bacterial cells is not well understood. However, it has been proposed that plant extracts induce bacterial cell death through the interaction of antimicrobial components of the plant extracts with enzymes and proteins of the microbial cell membrane. Hence disrupting microbial cell membrane to disperse high amounts of protons towards the cell exterior which induces cell death [82, 83]. Alternatively, it has been thought that hydrophobicity characters of plant extracts react with proteins of microbial cell membrane and mitochondria which affects their permeability resulting in cell death [30, 84]

None of the extracts or combinations with antifungal activity (AALM, SGLM, SGSM, AASM, AASW) had fungicidal activity against *C. albicans*.

#### Total antibacterial activity and total antifungal activity (TAA)

The total antimicrobial activity of the extracts is a pharmacologically useful measure to compare the antimicrobial efficacy of different plants since it takes into account both the anti-microbial activity and the quantity extracted from the plant material. The total activity of the extract is expressed in ml/g and is the volume of solvent that can be added to the extract obtained from 1 g of plant material that will still inhibit the growth of the specific pathogen [85]. Generally, the TAA of the extracts was highest when tested against *S. aureus,* followed by *E. coli* and least with *C. albicans* as shown in Table 9. AASM had the highest TAA against *S. aureus* (33.04 × 10^0^ ml/g)*, P. aeruginosa* (1.35 × 10^–1^ ml/g) and *C. albicans* (2.71 × 10^−4^ ml/g)*.* AALM had the highest TAA (1.13 × 10^0^ ml/g) against *E. coli* as shown in Table 9. Based on the TAA, *A. abyssinicus* leaves and stem bark powder had a high concentration of antimicrobial compounds and would require a high volume of solvent to dissolve it before it loses its antimicrobial activity compared to the *S. globulifera*. Therefore, *A. abyssinicus* stem bark would be the best choice for the treatment and development of drugs for ailments caused by *S. aureus* and *P. aeruginosa*. Elisha and collegues [85] report higher TAA values for *Bolusanthus speciosus*, *Calpurnia aurea*, *Maesa lanceolata*, *Hypericum roeperianum* and *Cremaspora triflora* against *E. coli* compared to those of *S. globulifera* and *A. abyssinicus* reported by this study. TAA of plant extract against any pathogen significant for publication should at least be 100 ml/g [39], however all TAA values of the current study were all below 100 ml/g hence not significant. However, they inform us on the variable overall antimicrobial activity of the leaves and stem bark of *S. globulifera* and *A. abyssinicus.*

**Table 9:** Total antimicrobial activity of *Symphonia globulifera* and *Allophylus abyssinicus* extracts

**Extract**	**TAA (ml/g)**			
	***Staphylococcus*** ***aureus***	***Escherichia*** ***coli***	***Pseudomonas*** ***Aeruginosa***	***Candida*** ***albicans***
AALW	7.08 × 10^0^	9.06 × 10^–1^	1.16 × 10^–1^	N/A
AALM	8.83 × 10^0^	1.13 × 10^0^	3.59 × 10^–2^	7.17 × 10^–5^
AASW	18.34 × 10^0^	5.82 × 10^–1^	7.45 × 10^–2^	1.49 × 10^–4^
AASM	33.04 × 10^0^	5.29 × 10^–1^	1.35 × 10^–1^	2.71 × 10^–4^
SGLW	11.83 × 10^0^	7.57 × 10^–1^	2.40 × 10^–2^	N/A
SGLM	2.04 × 10^0^	8.15 × 10^–3^	2.59 × 10^–4^	3.00 × 10^–7^
SGSM	5.56 × 10^0^	2.22 × 10^–2^	7.06 × 10^–4^	7.00 × 10^–7^
SGSW	10.16 × 10^0^	8.13 × 10^–2^	6.51 × 10^–4^	N/A

Key: *N/A* No Antifungal Activity, *SGLW Symphonia globulifera* leaf water extract, *SGLM Symphonia globulifera* leaf methanol extract, *SGSW Symphonia globulifera* stem bark water extract, *SGSM Symphonia globulifera* stem bark methanol extract, *AALW Allophylus abyssinicus* leaf water extract, *AALM Allophylus abyssinicus* leaf methanol extract, *AASW Allophylus abyssinicus* stem bark water extract, *AASM Allophylus abyssinicus* stem bark methanol extract

#### Fractional inhibitory concentration indices

The selected *S. globulifera* and *A. abyssinicus* extract combinations observed in the current study had antagonistic, indifferent, additive and synergistic interactions against *S. aureus, E. coli* and *P. aeruginosa* as shown in Table 10. *Allophylus abyssinicus* leaves water extract and *S. globulifera* leaves methanol extract combination showed a synergistic effect (ΣFICI = 0.3739) against *S. aureus.* Synergism arises as a result of mutual interaction between the extracts in that each enhances the activity of the other or as a result of a reaction between the two extracts forming a new component with a much high activity against the pathogen [86]. Furthermore, the combination of plant extracts increases the diversity of bioactive compounds which increases the number of drug targets that the combination can attack hence strong synergistic antimicrobial activity [87]. Interestingly extract combinations with AASW i.e. (AASW and AALW, SGLM and AASW, SGLW and AASW) had an additive combination effect against *S. aureus* which suggests that AASW could have a contributory effect in enhancing the potency and the activity of other extracts against *S. aureus.*

**Table 10:** Fractional inhibitory concentration indices of extract combinations of *Symphonia globulifera* and *Allophylus abyssinicus* against *Staphylococcus aureus*, *Escherichia coli* and *Pseudomonas aeruginosa*

Microorganism	Extract 1	Extract 2	FIC (1)	FIC (2)	ΣFIC	Combination Effect
*Staphylococcus aureus*	SGLW	AALW	4	2	6	Antagonistic
	SGLW	SGLM	1	0.2478	1.2478	Indifferent
	AASW	AALW	0.5	0.25	0.75	Additive
	SGLM	AASW	0.1239	0.5	0.6239	Additive
	SGLW	AASW	0.5	0.5	1	Additive
	AALW	SGLM	0.25	0.1239	0.3739	synergistic
*Escherichia* *coli*	SGLW	AALW	2	4	6	Antagonistic
	SGLW	SGLM	1	0.0625	1.0625	Additive
	AASW	AALW	0.2478	1	1.2478	Indifferent
	SGLM	AASW	0.0625	0.4957	0.5582	Additive
	SGLW	AASW	1	0.4957	1.4957	Indifferent
	AALW	SGLM	2	0.0625	2.0625	Indifferent
*Pseudomonas aeruginosa*	SGLW	AALW	0.2478	1	1.2478	Indifferent
	SGLW	SGLM	0.9914	0.9914	1.9828	Indifferent
	AASW	AALW	0.5	0.5	1	Additive
	SGLM	AASW	0.4957	2	2.4957	Indifferent
	SGLW	AASW	0.4957	2	2.4957	Indifferent
	AALW	SGLM	8	1.9828	9.9828	Antagonistic

Combination Scale ΣFIC =  ≤ 0.5:- Synergistic, > 0.5–1:-Additive, > 1–4:- Indifferent/Non interactive, > 4:-Antagonistic (Van Vuuren & Viljoen, 2011) [41]

*SGLW Symphonia globulifera* leaf water extract, *AALW Allophylus abyssinicus* leaf water extract, *SGLM Symphonia globulifera* leaf methanol extract, *AASW Allophylus abyssinicus* stem bark water extract

Both extract combinations SGLW and SGLM, SGLM and AASW had an additive effect against the growth of *E. coli* indicative of extract mutually exclusive antimicrobial activity against the bacteria with no chemical interaction between the extracts [41]. Focusing on *P. aeruginosa*, AASW and AALW combination had an additive effect with the lowest fractional inhibitory concentration index (ΣFICI = 1) against *P. aeruginosa* and all other combinations had either antagonistic or indifferent activity. Plant extract combination studies [88–90] mainly assume that each extract is a single bioactive compound yet the phytochemical composition of any plant extract is naturally diverse with high chemical complexity hence a range of pharmacological effects [91, 92]. It is worth noting that revelations of synergy, additive, non-interactive and antagonism are all equally important in phytotherapy. Plant extract combinations with synergistic and additive effects can be chosen for the development of combination phytotherapy.

## Conclusion

*Symphonia globulifera* and *Allophylus abyssinicus* stem bark and leaves contain a wide range of bioactive compounds with antimicrobial activity for example tannins, alkaloids, flavonoids, quinones, anthraquinones among others. However, antimicrobial assays proved that the water and methanolic crude extracts of both *S. globulifera* and *A. abyssinicus* stem bark and leaves had weak antimicrobial activity against *S. aureus*, *E. coli, P. aeruginosa and C. albicans,* but the extracts have a higher antibacterial activity than antifungal activity. Interestingly, the leaves of both plants had higher total antibacterial activity (TAA) than the stem bark, therefore leaves rather than the stem bark should be utilized for large-scale development of phyto-remedies from these plants. The current study has only offered a preliminary phytochemical profile within the plant extracts, we therefore recommend further studies using methods like liquid chromatography-mass spectrometry for isolation, and identification of different compounds. Last but not least, in vivo antimicrobial activity of these extracts and phytochemical compounds present in the extracts of the *S. globulifera* and *A. abyssinicus* would offer more insight into understanding their bioactivity.

## Data Availability

The datasets used and/or analysed during the current study are available from the corresponding author on reasonable request.
